# THE ROLE OF LATE-LIFE WORK AMONG KOREAN WIDOWED ADULTS

**DOI:** 10.1093/geroni/igad104.2510

**Published:** 2023-12-21

**Authors:** Hyeonji Cho, Nahyun Kim, Kyungmin Kim

**Affiliations:** Seoul National University, Seoul, Republic of Korea; Seoul National University, Seoul, Republic of Korea; Seoul National University, Seoul, Republic of Korea

## Abstract

According to the Dual Process Model, oscillation between two types of stressors and coping processes—loss-oriented and restoration-oriented leads bereaved individuals toward the adaptive coping. Previous studies have focused on the buffering effects of social engagement such as volunteering or leisure as restoring activities, but little is known about the role of work. In Korea, employment rates of older adults are rising, and many people wish to keep working after the retirement age. This study examines the restoring effects of work among Korean widowed adults. Utilizing five waves of the Korean Retirement and Income Study (2011–2019; conducted biennially), we analyzed 481 middle-aged and older adults (aged 52–95) who experienced spousal loss during the study period. The widowed participants provided data on their employment status and perceived health status, including sociodemographic characteristics at both pre-loss (T1) and post-loss (T2). On average, participants’ perceived physical health (MT1 = 2.75, MT2 = 2.61) and mental health (MT1 = 3.20, MT2 = 3.07) deteriorated after the bereavement. Residualized change models found that employed participants (29.7%) showed a smaller decrease in perceived physical health (B = 0.35, p < .001) and mental health (B = 0.18, p = .046) compared to their counterparts, which supports the buffering effect of late-life work in the bereavement process. We also found a significant interaction between employment status and net wealth for perceived physical health (B = -0.04, p = .037), showing that the effect of employment stood out more among economically vulnerable people.

